# Giant primary adrenal hydatid cyst presenting with arterial hypertension: a case report and review of the literature

**DOI:** 10.1186/1752-1947-6-46

**Published:** 2012-02-01

**Authors:** Fadl Tazi, Mustapha Ahsaini, Abdelhak Khalouk, Soufiane Mellas, Roos E Stuurman-Wieringa, Mohammed Jamal Elfassi, Moulay Hassan Farih

**Affiliations:** 1 Department of Urology, Hospital University Center Hassan II, 30000 Fez, Morocco; 2 Department of Urology, Academic Medical Center, PO Box 22660, 1100 DD, Amsterdam, Netherlands

## Abstract

**Introduction:**

A primary hydatid cyst of the adrenal gland is still an exceptional localization. The adrenal gland is an uncommon site even in Morocco, where echinococcal disease is endemic.

**Case presentation:**

We report the case of a 64-year-old Moroccan man who presented with the unusual symptom of arterial hypertension associated with left flank pain. Computed tomography showed a cystic mass of his left adrenal gland with daughter cysts filing the lesion (Type III). Despite his negative serology tests, the diagnosis of a hydatid cyst was confirmed on surgical examination. Our patient underwent surgical excision of his left adrenal gland with normalization of blood pressure. No recurrence has occurred after 36 months of follow-up.

**Conclusion:**

There are two remarkable characteristics of this case report; the first is the unusual location of the cyst, the second is the association of an adrenal hydatid cyst with arterial hypertension, which has rarely been reported in the literature.

## Introduction

Hydatid disease (HD) is caused by *Echinococcus granulosus *larva and is endemic in many countries, including those in the Middle East, Eastern Europe, Africa, Latin America and China. It poses an important public health problem and disease spread is influenced by socioeconomic status and migration [1]. Although rare, it may occur in any organ or tissue. The most common site is the liver (59% to 75%), followed in frequency by the lung (27%), kidney (3%), bone (14%) and brain (12%) [2]. Other sites, such as the heart, spleen, pancreas and muscles, are very rarely affected.

The adrenal gland is considered as an exceptional localization of a hydatid cyst. Parasitic cysts involving the adrenal glands are usually secondary and part of generalized echinococcosis. The reported incidence of adrenal hydatidosis is 0.5% [3]. We report the case of a 64-year-old man who presented with a vague abdominal pain in his left flank, with a recent history of arterial hypertension. Our patient underwent surgical excision of his adrenal gland, with normalization of his blood pressure three months later.

## Case presentation

A 64-year-old Moroccan man was admitted to our department for left flank pain with no particular irradiation that had started one month previously and nausea and vomiting of one day's duration. He reported no digestive or urinary symptoms and was afebrile. He had no significant medical history apart from new-onset arterial hypertension one year before (systolic blood pressure, 170 mmHg to 190 mmHg; diastolic blood pressure, 90 mmHg to 100 mmHg) requiring combination therapy with bisoprolol and amlodipine. He denied any contact with dogs or sheep.

**Figure 1:**
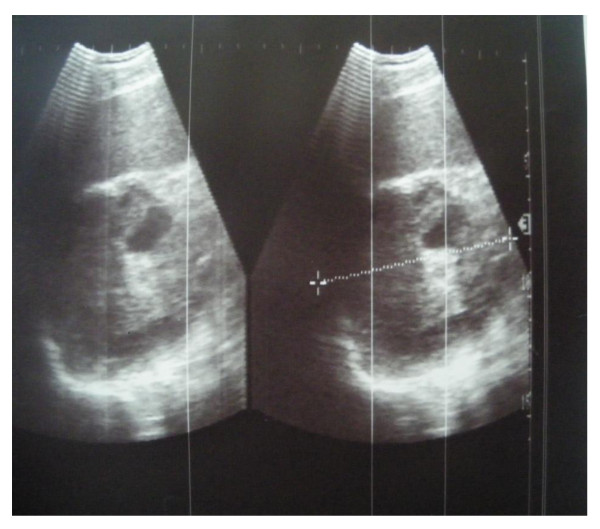
**Abdominal ultrasonography revealing a large hypoechoic mass, measuring 10.4 cm in the largest diameter, in the upper pole of his left kidney**.

**Figure 2:**
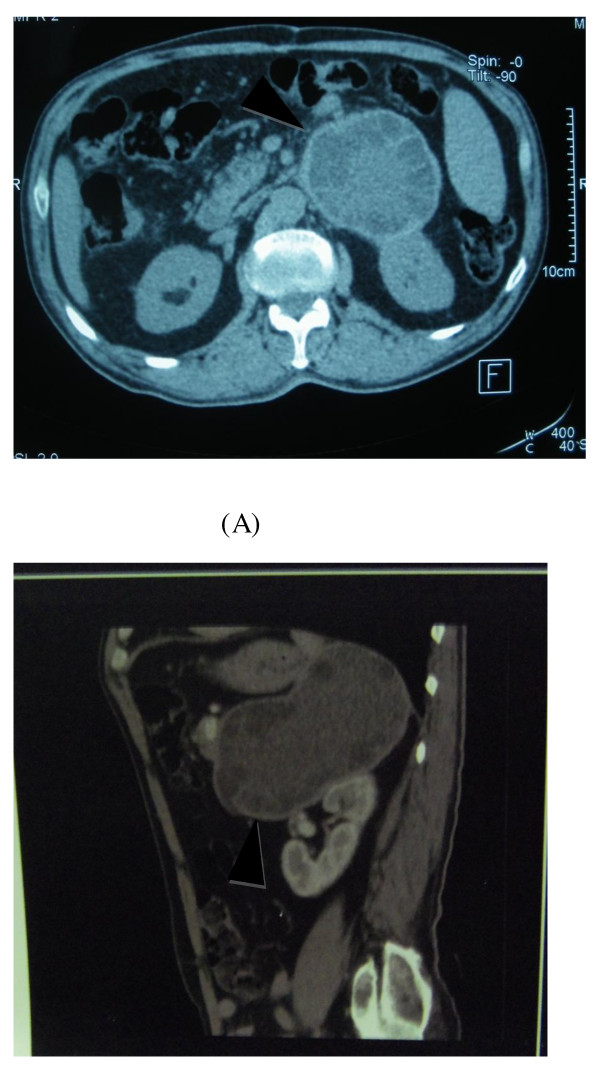
**Type III hydatid cyst**. Computed tomography scans show daughter cysts filing the lesion, which has a honeycomb appearance.

His physical examination was unremarkable and a standing abdominal radiography was normal. Abdominal ultrasonography revealed a large hypoechoic mass, containing an internal cystic component adjacent to the superior pole of his left kidney. Initially it was almost impossible to find its contacts and origin (Figure 1). A computed tomography (CT) scan of his abdomen showed a giant hypoattenuated cystic mass in the top of his left kidney measuring 14.5 cm × 7.2 cm with daughter cysts filing the lesion, which had a honeycomb appearance in his left adrenal gland, with a mass effect on surrounding structures (Figure 2A, B). An exploration of the rest of his abdominal cavity and chest didn't find any further evidence of HD, including his liver and lungs. A hematological examination was characterized by a slight augmentation of white blood cells (11 cells/mm^3^) and by eosinophilia (7% eosinophils). His blood biochemistry was normal. Other laboratory results were normal, including his renin, aldosterone and 24-hour urinary vanillylmandelic acid and metanephrine levels. A serologic test of the hydatid cyst was negative.

After the radiological findings, the mass was diagnosed as a giant primary adrenal hydatid cyst; although our patient denied animal contact, the cyst was presumed to be hydatid, and surgical exploration was planned, based on these data.

Our patient underwent an open laparotomy through a midline incision and the cystic lesion of his left adrenal gland was identified. The exploration found a multivesicular hydatid cyst of the upper pole of the left adrenal measuring 18 cm×10 cm×8 cm. The abdominal cavity and especially the region around the cyst was carefully protected and isolated by pads soaked in hypertonic 20% saline solution. The cyst was not drained nor aspirated during the surgical procedure. An appropriate dissection plane between the cyst and the adrenal gland could not be found, so both the cyst and the adrenal gland were removed. It is noteworthy that our patient remained hemodynamically stable without any hypertensive crisis during the operation. A macroscopic examination revealed a germinate membrane and daughter cysts and a microscopic examination revealed scolices in the cyst fluid. A histological examination confirmed the diagnosis. The postoperative course was uneventful. Our patient underwent therapy with albendazole (800 mg/day) for one month. There was a progressive reduction in his arterial pressure after the operation, and three months after the surgery his blood pressure was about 130/80 mmHg, with no medication and no particular diet. At the most recent follow-up, 24 months later, ultrasound monitoring did not detect any hydatid recurrence, and our patient was doing well and symptom free.

## Discussion

Symptomatic hydatid cystic disease of the adrenal glands is rarely observed in clinical practice and accounts for about 7% of the cysts in the adrenal gland [4]. Most hydatid cysts arising from the adrenal gland are part of a disseminated disease and a primary adrenal hydatid cyst is very unusual [4]. The mechanism of the adrenal involvement is not clear. The most likely theory is that an embryo of *E. granulosus *passes through lung and liver filters to disseminate through the bloodstream. There have been 20 cases of primary hydatid cyst of the adrenal glands reported up to 2007 [5]. Most adrenal hydatid cysts are asymptomatic and found incidentally by imaging or during surgery for other abdominal pathologies [6], or the symptoms resulted from space-occupying lesions [7], such as the vague abdominal and flank pain in our patient, gastrointestinal complaints or a palpable mass [4]. Rarely, endocrine abnormalities like arterial hypertension are seen in adrenal HD [8-11]. Clinical symptoms suggestive of a pheochromocytoma have also been reported, which are related to the compression of the adrenal medulla by the cyst [12].

Our reported case presents two important elements. Firstly, primary HD of the adrenal gland is rare and only a few cases have been reported in the literature [5]. Secondly, our patient underwent surgical excision of his adrenal gland with a normalization of arterial hypertension. Our patient had a one-year history of arterial hypertension. Until now, a few cases with the coexistence of a primary adrenal hydatid cyst and arterial hypertension have been reported in the literature [8-11]. However, there is no clear and acceptable explanation about the relationship and the involved pathogenetic mechanism; a possible explanation is the Goldblatt phenomenon of hypertension resulting from partial occlusion of a renal artery by external pressure [13]. High blood pressure appears to be a multifactorial disorder in which the interaction of several components with each other and with the environment is important [10].

In laboratory tests, eosinophilia is present in 25% of cases, as in our patient. A Casoni skin test and indirect hemagglutination tests are not completely reliable for diagnosis. New, more sensitive and specific serological tests are available, including complement fixation, enzyme-linked immunosorbent assays, arc-5 precipitation and a specific hydatid immunoglobulin E test [6], which can provide diagnostic certainty when positive. The sensitivity of this latter test is 90%, but if it is negative the cause of a hydatid cyst of the adrenal gland can't be eliminated, like our patient who had a negative test.

The imaging features of HD depend on the stage of evolution of the disease. Early lesions appear purely cystic; after modification of the germinal layer and a reduction of intraluminal pressure the capsule becomes fibrotic and sometimes calcified, while the daughter cysts discharge from the wall and float in the lumen.

Hydatid cyst identification in the adrenal gland is based mainly on ultrasonography and CT scans [14], with the diagnostic sensitivity of ultrasonography ranging from 93% to 98%, while that of CT is around 97% [15]. On radiology, hydatid cysts can be described according to the classification of Gharbi *et al. *[16], which is based on ultrasonography features and includes five types. Type I is a well-defined, anechoic lesion. Type II demonstrates separation of the membrane; the 'water lily' sign is formed by the undulating membrane. Type III is characterized by septa and intraluminal daughter cysts. Type IV is a nonspecific solid mass. Type V is a solid mass with a calcified capsule that can be identified by abdominal X-ray as a calcification of the cystic wall. In our case, the adrenal hydatid cyst was a type III according to the classification of Gharbi *et al*.

Few reports of magnetic resonance imaging (MRI) of adrenal HD have been published. On MRI, the complex cyst contents can be well displayed and the cyst membrane, whether collapsed or not, can be clearly seen as a low-intensity curvilinear structure on both short and long repetition time spin-echo images [17].

The differential diagnosis of an adrenal cyst should include an endothelial cyst, a pseudocyst due to infarction or hemorrhage in the adrenal gland, cystic neoplasm and a post-traumatic cyst [7,18,19].

Treatment of HD of the adrenal glands is mostly surgical. Pericystectomy and resection of the entire adrenal gland are the two preferred choices. Resection of the cyst with conservation of the gland remains the optimal procedure. It is preferable to follow an appropriate dissection plane between the cyst and the adrenal gland in order for a complete cystectomy to be achieved. However, it is not always feasible and may cause hemorrhage owing to the close adherence of the cystic wall to the adrenal gland's parenchyma. In case of hemorrhage or a failure to perform the cystectomy, ablation of the entire adrenal gland including the cyst should be performed. Partial cystectomy includes a higher risk of dissemination of the parasite due to the dissection's maneuvers [9]. Access to the adrenal gland may be through an anterior transabdominal or posterior retroperitoneal traditional open approach. Cyst injection with hypertonic saline solution before puncture can inactivate scolices and daughter cysts [6]. In our patient, an anterior subcostal transperitoneal approach was used and cyst fluid aspiration and hypertonic saline solution injection into the sac preceded attempts to remove the cyst.

Recent minimally invasive surgical techniques like transabdominal laparoscopy and retroperitoneal endoscopic adrenalectomy have been conducted [20,21]. However, despite the benefits of this technique, like a lower morbidity and avoidance of the major laparotomy incision necessary in open procedures [22], many authors do not recommend this approach for complex cysts [23] and warn that cysts larger than 8 cm in diameter have a higher risk of spillage of daughter cysts and development of secondary HD [24]. Moreover, it has been shown that an open approach has the advantage of allowing the surgeon to explore the peritoneal cavity [10].

Puncture, aspiration, injection and re-aspiration (PAIR) treatment of hydatidosis is considered minimally invasive, confirms the diagnosis and improves the efficacy of chemotherapy given before and after puncture. PAIR is usually avoided in patients with adrenal hydatidosis, but can become an alternative method in inoperable cases [13]; however, many authors contraindicate its use because of potential complications, such as anaphylactic shock and the spread of daughter cysts [25].

Antihelmintic agents have been used in the treatment of systemic echinococcosis in endemic areas [25]. There are reports that antihelmintic agents can reduce the size of cysts in some cases, however the results are not satisfactory and this treatment should be limited for disseminated and recurrent cysts or in cases where surgery is contraindicated [25]. In our patient, although any obvious spillage had not been recorded, one month of oral albendazole 800 mg/day was started postoperatively to avoid recurrence because of the large size of the cyst.

## Conclusion

In endemic countries such as Morocco, primary HD should be considered in the differential diagnosis of an adrenal cyst and no attempt to preoperatively puncture or to perform any other manipulation of the lesion should be done. CT associated with hydatid serology can confirm the diagnosis. The treatment of choice is a pericystectomy of the hydatid cyst if it is possible or, otherwise, total excision of the adrenal gland. The association of an adrenal hydatid cyst with hypertension is very rare. In such cases, surgical removal of the hydatid cyst may also treat the hypertension. Laparoscopic resection of an adrenal hydatid cyst is an alternative to conventional surgery; however, it has been described only sporadically. Finally, the prevention of hydatid infection is a necessary step to prevent HD regardless of its location.

## Consent

Written informed consent was obtained from the patient for publication of this case report and any accompanying images. A copy of the written consent is available for review by the Editor-in-Chief of this journal.

## Competing interests

The authors declare that they have no competing interests.

## Authors' contributions

FT and MA were the principal authors and major contributors in writing the manuscript. AK and SM analyzed and interpreted the patient data and reviewed the literature. RE, SW, MJE and MHF read and corrected the manuscript. All authors read and approved the final manuscript.
